# Health Questions Posed by Amerindians in Guyana’s Deep Interior

**DOI:** 10.5539/gjhs.v4n6p43

**Published:** 2012-08-22

**Authors:** Raywat Deonandan, Bekkie Vineberg, Rebecca Stulberg, Marya Jaleel, Nicholas Szecket, Sheila Dunn, Mary Ann Hamelin, Margaret Van der Kuur, Sarah Zelcer

**Affiliations:** 1 Interdisciplinary School of Health Sciences, University of Ottawa, Ottawa, Canada; 2 McMaster University, Hamilton, Canada; 3 Ve’ahavta, Toronto, Canada; 4 Women’s College Research Institute, Women’s College Hospital, Toronto, Canada

**Keywords:** global health, aboriginal, epidemiology, reproductive health

## Abstract

**Background:**

The forest-dwelling Amerindian peoples of Guyana are among that nation’s most impoverished, vulnerable and least served. Health promotion messaging has been informed in large part by nation-level health indicators that may not be well targeted to this group. Our study sought to identify local health education needs, and to identify factors preventing proper uptake of health messaging.

**Methods:**

As part of medical missions to the interior, we asked patients waiting for care to anonymously submit their health questions in writing. Conventional content analysis was employed to identify prevalent themes in their responses.

**Findings:**

Sexual health (63.6%) and nutrition (17.4%) were the most popular themes asked about. Within the former, the science of sexual maturation and reproduction (31.4%) and HIV/AIDS (28.8%) were the most common sub-themes, with the pathophysiology and etiology of HIV/AIDS being the most common sub-theme within the latter.

**Interpretation:**

Within Guyana’s Amerindian community, there exists a prevalent curiosity about the basic science of both sexual reproduction and the transmission of sexual disease.

## 1. Introducation

In many countries, aboriginal populations are vulnerable to environmental and societal changes, leading to health crises within their communities. An excellent example of this is seen the marginalized Amerindian communities in the Mazaruni region of Guyana, South America, and its immediate neighbours (Mantini et al., 2008; Peplow et al., 2012). Guyana is a poor, English-speaking country on the South American continent, but with economic and cultureal affiliations with the nations of the Caribbean. It is underpopulated, with fewer than 800,000 residents, though almost all are clustered along the Caribbean coast.

The interior of the country is largely undeveloped rainforest, sparsely populated by AmerIndian tribes, and of interest to international mining and timber concerns. These communities, made up in large part of Awarak tribes, consist of villages of 200-300 people situated on the banks of rivers within the South American rainforest. Their economies have been largely re-tasked to service the mining industry, which, anecdotal and some empirical evidence suggests, has resulted in an increase in migrant labour, environmental toxins, diabetes, obesity and sexually transmitted diseases (Frery et al., 2001; Seguy et al., 2008). In particular, HIV/AIDS is thought to be a serious concern in these populations (Palmer et al., 2002), with a national prevalence rate of 2.4% (Government of Guyana, 2012). This has come in partnership with a decrease in local food production and an ongoing crisis in health literacy and health care resourcing.

Direct health care interventions in such remote communities are unavoidably costly, requiring the transportation of people and equipment hundreds of miles through dense rainforest. Funding priorities, therefore, have focused on the needs of the more accessible, coastal-dwelling populations. Priorities have traditionally included HIV/AIDS, maternal and child health, diabetes and nutrition (Pan American Health Organization, 2001). The interior-dwelling Amerindian population has not received a great deal of funding attention nor has it been a consistent target for epidemiological investigation of health need, despite being the most impoverished sector of Guyanese society (Pan American Health Organization, 2001).

Given the lack of targeted population health information (Ministry of Health of Guyana, 2008), the needs assessments defining extant limited investments in Amerindian health have likely been informed by country-level indicators, which may or may not be reflective of the actual local needs of the forest-dwelling population. The Toronto-based NGO Ve’ahavta has sent regular medical missions into Amerindian communities in Guyana’s Mazaruni region for several years. During two such missions, residents in the villages of Kamarang and Waramadong were given the opportunity to anonymously ask questions about personal and community health issues. These questions constitute a qualitative community-based health needs assessment, separate from the standard population-level indicators that typically inform health policy for these populations.

This paper describes a summary, by theme, of the health questions posed by the villagers, organized to suggest an alternative, ground-up health needs assessment for this and similar aboriginal populations

## 2. Method

During two visits by Ve’ahavta ‘s medical volunteers, once in March of 2008 and again in March of 2010, Amerindians who had gathered to receive direct clinical care from Canadian doctors were given a standard group health education presentation, focusing on sexual health and personal hygiene. In the course of presentation, a bag with pieces of paper with pens were passed around the group, and the attendees were instructed to anonymously write down any questions about any aspects of their personal or community health that they wished the health educator to address to the group, and to place their questions in the bag.

In 2008, both Kamarang and Waramadong were visited. In 2010, only Waramadong was visited, due to scheduling limitations. The presentation and instructions were given in the local vernacular. Selected responses were taken from the bag and informed the content of the remaining presentation time. In Waramadong, the medical teams’ visits to the local residential public school contributed significantly to the complement of responses. All of the written questions were later qualitatively analyzed using conventional content analysis, as described by Hsieh and Shannon (2005), to determine prevalent themes and sub-themes.

Ethics approval for this study was granted by the University of Ottawa research ethics office.

## 3. Results

In this paper, we refer to the questions offered by subjects as “responses”. Typically, 100-300 patients would gather at any of our clinics. In 2008, 33 responses were gathered from the Kamarang visit, while 66 were received in Waramadong. In 2010, 98 responses were collected in Waramadong. Several responses featured more than question, or were coded as being relevant to more than one content theme. Respondents were a combination of village residents, residential teenaged schoolchildren and residents of surrounding villages who had journeyed to both locations specifically to access medical care.

Six major themes arose from our content analysis: Diabetes, Alcohol/drugs/smoking, Heart disease/hypertension/atherosclerosis, Malaria, Nutrition, and Sexual Health. A Miscellaneous category was created for 16 responses, which included, in addition to other issues, questions relating to hair loss, stress, and cancer. The distribution of responses across the major thematic areas is summarized in Table 1.

**Table 1:** Subjects’ overall response count according to theme, year and location

Theme	Response count by location and year			Total	Percent distribution

	Kamarang 2008	Waramadong 2008	Waramadong 2010		
Diabetes	4	0	4	8	3.2%
Alcohol/drugs/smoking	7	7	10	24	9.7%
Heart disease/hypertension/atherosclerosis	5	0	4	9	3.6%
Malaria	0	1	5	6	2.4%
Nutrition	7	23	13	43	17.4%
Sexual Health	25	66	66	157	63.6%
Total	48	97	102	247	100%

Given that Sexual Health garnered much interest, its responses were given a deeper content analysis. The distribution of Sexual Health responses across sub-themes is presented in Table 2.

**Table 2:** Subjects’ response count for sub-themes of sexual health, according to year and location

Sexual Health Sub-theme	Response count by location and year			Total	Percent distribution

	Kamarang 2008	Waramadong 2008	Waramadong 2010		
Science of sexual maturation and reproduction	11	26	12	49	31.4%
Physiology of reproductive organs	1	7	3	11	7.1%
Practising safe sex	7	9	4	20	12.8%
Birth control	5	6	4	15	9.6%
Prevention/treatment of STIs	0	8	7	15	9.6%
HIV/AIDS	1	8	36	45	28.8%
Family planning	0	1	0	1	Negligible
Total	25	65	66	156	100%

Responses to the HIV/AIDS sub-theme were further broken down into three categories: the pathophysiology or etiology of the disease, prevention or treatment of HIV/AIDS, and signs/symptoms of infection. The distribution of those responses is presented in Table 3.

**Table 3:** Response count for sub-themes of HIV/AIDS

HIV/AIDS Sub-Theme	Number of Responses	Percent distribution
Pathophysiology/Etiology	33	73.3%
Prevention/Treatment	7	15.6%
Signs/symptoms	5	11.1%
Total	45	100%

## 4. Discussion

At face value, the predominance of Sexual Health, in particular HIV/AIDS, in our content analysis suggests that villagers’ concerns are recapitulating the national funding trends, which also focus on reproductive and sexual health issues, lending validity to the official health funding policies. However, one must consider three sources of bias implicit in our approach. The first is the oversampling of teenaged residential students in Waramadong, for whom sexual issues are a natural point of interest, given their age.

The second is the unavoidable ubiquitous messaging around HIV/AIDS that percolates all about Guyana, due to persistent government literature, NGO missions, and health promotion efforts in the form of traveling “street” theatre, posters in the health stations and advertisements on radio. This continuously reinforces the perceived importance of HIV/AIDS to population and individual health.

The third source of bias is the exclusive sampling of individuals sufficiently literate in English to write questions legibly. There was a sense that those most likely to be insufficiently English literate were the extremely elderly, whose first language was more likely to be a tribal dialect. However, during visits to the schools of Waramadong, all of the respondents were highly literate. How this bias affects our insights is uncertain, except to the extent that is unlikely that the extremely elderly would be as concerned with Sexual Health.

Despite these biases, it is telling that most of the curiosity concerning HIV/AIDS is not about its prevention or treatment, which is where most interventionist programs tend to focus, but on its pathophysiological essence. Sample responses in this domain include, “What is AIDS?” (a common question) and “What is the difference between HIV and AIDS?” This suggests a fundamental educational gap about the nature of the disease that must be filled before prevention campaigns can achieve full potency.

The other popular sub-theme of Sexual Health was the basic science surrounding reproductive physiology and maturation. Questions such as, “Why do I sometimes get my period twice a month?” and “Can a girl get pregnant if she has unprotected sex during menstruation?” suggest, again, that a gap in basic science education is a barrier to proper uptake of public health messaging.

Given that this population, like much of the global Aboriginal population, suffers disproportionately from Diabetes. Questions like, “What is a carbohydrate?” suggest that previous public health messaging has employed a largely didactic approach, without sufficiently accounting for the appropriateness of language and the need to provide basic nutritional science overviews.

It is a surprising result that there was little curiosity expressed about either Tuberculosis or Malaria. Both are serious health issues in Guyana (Alladin et al., 2011; Rawlins et al., 2008), though Malaria is on the decline. Considerations of our aforementioned biases aside, this suggests either that long term multi-generational experience with these diseases is sufficient to quell much curiosity, or that these diseases are not considered to be as important or as interesting subjects as are sexual issues. If indeed this is the result of lack of appreciation of the prevalence of TB, in particular, then it is concerning that health promotion efforts have perhaps oversold the importance of Sexual Health at the expense of self-protection from other infectious diseases.

Our qualitative approach leads to insights that are, of course, purely speculative and suggestive, not conclusive. Our results suggest that a more structured, quantitative study of the health literacy and personal health curiosities of Amerindian peoples would help to better inform the plethora of health promotion projects currently besetting these populations.
